# Challenges and Opportunities with Causal Discovery Algorithms: Application to Alzheimer’s Pathophysiology

**DOI:** 10.1038/s41598-020-59669-x

**Published:** 2020-02-19

**Authors:** Xinpeng Shen, Sisi Ma, Prashanthi Vemuri, Gyorgy Simon, the Alzheimer’s Disease Neuroimaging Initiative, Michael W. Weiner, Paul Aisen, Ronald Petersen, Clifford R. Jack, Andrew J. Saykin, William Jagust, John Q. Trojanowki, Arthur W. Toga, Laurel Beckett, Robert C. Green, John Morris, Leslie M. Shaw, Zaven Khachaturian, Greg Sorensen, Maria Carrillo, Lew Kuller, Marc Raichle, Steven Paul, Peter Davies, Howard Fillit, Franz Hefti, David Holtzman, M. Marcel Mesulam, William Potter, Peter Snyder, Adam Schwartz, Tom Montine, Ronald G. Thomas, Michael Donohue, Sarah Walter, Devon Gessert, Tamie Sather, Gus Jiminez, Archana B. Balasubramanian, Jennifer Mason, Iris Sim, Danielle Harvey, Matthew Bernstein, Nick Fox, Paul Thompson, Norbert Schuff, Charles DeCArli, Bret Borowski, Jeff Gunter, Matt Senjem, David Jones, Kejal Kantarci, Chad Ward, Robert A. Koeppe, Norm Foster, Eric M. Reiman, Kewei Chen, Chet Mathis, Susan Landau, Nigel J. Cairns, Erin Franklin, Lisa Taylor-Reinwald, Virginia Lee, Magdalena Korecka, Michal Figurski, Karen Crawford, Scott Neu, Tatiana M. Foroud, Steven Potkin, Kelley Faber, Sungeun Kim, Kwangsik Nho, Leon Thal, Neil Buckholtz, Marilyn Albert, Richard Frank, John Hsiao, Jeffrey Kaye, Joseph Quinn, Lisa Silbert, Betty Lind, Raina Carter, Sara Dolen, Lon S. Schneider, Sonia Pawluczyk, Mauricio Beccera, Liberty Teodoro, Bryan M. Spann, James Brewer, Helen Vanderswag, Adam Fleisher, Judith L. Heidebrink, Joanne L. Lord, Sara S. Mason, Colleen S. Albers, David Knopman, Kris Johnson, Rachelle S. Doody, Javier Villanueva-Meyer, Valory Pavlik, Victoria Shibley, Munir Chowdhury, Susan Rountree, Mimi Dang, Yaakov Stern, Lawrence S. Honig, Karen L. Bell, Beau Ances, Maria Carroll, Mary L. Creech, Erin Franklin, Mark A. Mintun, Stacy Schneider, Angela Oliver, Daniel Marson, David Geldmacher, Marissa Natelson Love, Randall Griffith, David Clark, John Brockington, Erik Roberson, Hillel Grossman, Effie Mitsis, Raj C. Shah, Leyla deToledo-Morrell, Ranjan Duara, Maria T. Greig-Custo, Warren Barker, Chiadi Onyike, Daniel D’Agostino, Stephanie Kielb, Martin Sadowski, Mohammed O. Sheikh, Anaztasia Ulysse, Mrunalini Gaikwad, P. Murali Doraiswamy, Jeffrey R. Petrella, Salvador Borges-Neto, Terence Z. Wong, Edward Coleman, Steven E. Arnold, Jason H. Karlawish, David A. Wolk, Christopher M. Clark, Charles D. Smith, Greg Jicha, Peter Hardy, Partha Sinha, Elizabeth Oates, Gary Conrad, Oscar L. Lopez, Mary Ann Oakley, Donna M. Simpson, Anton P. Porsteinsson, Bonnie S. Goldstein, Kim Martin, Kelly M. Makino, M. Saleem Ismail, Connie Brand, Adrian Preda, Dana Nguyen, Kyle Womack, Dana Mathews, Mary Quiceno, Allan I. Levey, James J. Lah, Janet S. Cellar, Jeffrey M. Burns, Russell H. Swerdlow, William M. Brooks, Liana Apostolova, Kathleen Tingus, Ellen Woo, Daniel H. S. Silverman, Po H. Lu, George Bartzokis, Neill R Graff-Radford, Francine Parfitt, Kim Poki-Walker, Martin R. Farlow, Ann Marie Hake, Brandy R. Matthews, Jared R. Brosch, Scott Herring, Christopher H. van Dyck, Richard E. Carson, Martha G. MacAvoy, Pradeep Varma, Howard Chertkow, Howard Bergman, Chris Hosein, Sandra Black, Bojana Stefanovic, Curtis Caldwell, Ging-Yuek Robin Hsiung, Benita Mudge, Vesna Sossi, Howard Feldman, Michele Assaly, Elizabeth Finger, Stephen Pasternack, Irina Rachisky, John Rogers, Dick Trost, Andrew Kertesz, Charles Bernick, Donna Munic, Emily Rogalski, Kristine Lipowski, Sandra Weintraub, Borna Bonakdarpour, Diana Kerwin, Chuang-Kuo Wu, Nancy Johnson, Carl Sadowsky, Teresa Villena, Raymond Scott Turner, Kathleen Johnson, Brigid Reynolds, Reisa A. Sperling, Keith A. Johnson, Gad Marshall, Jerome Yesavage, Joy L. Taylor, Barton Lane, Allyson Rosen, Jared Tinklenberg, Marwan N. Sabbagh, Christine M. Belden, Sandra A. Jacobson, Sherye A. Sirrel, Neil Kowall, Ronald Killiany, Andrew E. Budson, Alexander Norbash, Patricia Lynn Johnson, Thomas O. Obisesan, Saba Wolday, Joanne Allard, Alan Lerner, Paula Ogrocki, Curtis Tatsuoka, Parianne Fatica, Evan Fletcher, Pauline Maillard, John Olichney, Charles DeCarli, Owen Carmichael, Smita Kittur, Michael Borrie, T.-Y. Lee, Rob Bartha, Sterling Johnson, Sanjay Asthana, Cynthia M. Carlsson, Pierre Tariot, Anna Burke, Ann Marie Milliken, Nadira Trncic, Adam Fleisher, Stephanie Reeder, Vernice Bates, Horacio Capote, Michelle Rainka, Douglas W. Scharre, Maria Kataki, Brendan Kelly, Earl A. Zimmerman, Dzintra Celmins, Alice D. Brown, Godfrey D. Pearlson, Karen Blank, Karen Anderson, Laura A. Flashman, Marc Seltzer, Mary L. Hynes, Robert B. Santulli, Kaycee M. Sink, Leslie Gordineer, Jeff D. Williamson, Pradeep Garg, Franklin Watkins, Brian R. Ott, Geoffrey Tremont, Lori A. Daiello, Stephen Salloway, Paul Malloy, Stephen Correia, Howard J. Rosen, Bruce L. Miller, David Perry, Jacobo Mintzer, Kenneth Spicer, David Bachman, Nunzio Pomara, Raymundo Hernando, Antero Sarrael, Susan K. Schultz, Karen Ekstam Smith, Hristina Koleva, Ki Won Nam, Hyungsub Shim, Norman Relkin, Gloria Chaing, Michael Lin, Lisa Ravdin, Amanda Smith, Balebail Ashok Raj, Kristin Fargher

**Affiliations:** 1 Institute for Health Informatics, University of Minnesota, Minneapolis, MN 55455 USA; 2 Mayo Clinic, Rochester, MN 55905 USA; 3 University of California, San Francisco, USA; 4 University of Southern California, Los Angeles, USA; 5 Indiana University, Bloomington, USA; 6 University of California, Berkeley, Berkeley USA; 7 University of Pennsylvania, Philadelphia, USA; 8 University of California, Davis, Davis USA; 9 Brigham and Women’s Hospital/Harvard Medical School, Boston, USA; 10 Washington University St. Louis, St. Louis, USA; 11 Prevent Alzheimer’s Disease, 2020 Rockville, USA; 12 Siemens, Munich, Germany; 13 Alzheimer’s Association, Illinois, USA; 14 University of Pittsburgh, Pennsylvania, USA; 15 Cornell University, New York, USA; 16 Albert Einstein College of Medicine of Yeshiva University, New York, USA; 17 AD Drug Discovery Foundation, New York, USA; 18 Acumen Pharmaceuticals, California, USA; 19 Northwestern University, Illinois, USA; 20 National Institute of Mental Health, Maryland, USA; 21 Brown University, Rhode Island, USA; 22 Eli Lilly, Indiana, USA; 23 University of Washington, Washington, USA; 24 University of California, San Diego, California USA; 25 University of London, London, UK; 26 University of California, Los Angeles, California USA; 27 University of Michigan, Michigan, USA; 28 University of Utah, Utah, USA; 29 Banner Alzheimer’s Institute, Arizona, USA; 30 University of California, Irvine, California USA; 31 National Institute on Aging, Maryland, USA; 32 Johns Hopkins University, Maryland, USA; 33 Richard Frank Consulting, New Hampshire, USA; 34 Oregon Health and Science University, Oregon, USA; 35 Baylor College of Medicine, Texas, USA; 36 Columbia University Medical Center, New York, USA; 37 University of Alabama-, Birmingham, Alabama USA; 38 Mount Sinai School of Medicine, New York, USA; 39 Rush University Medical Center, Rush University, Illinois, USA; 40 Wien Center, Florida, USA; 41 NewYork University, New York, USA; 42 Duke University Medical Center, North Carolina, USA; 43 University of Kentucky, Kentucky, USA; 44 University of Rochester Medical Center, New York, USA; 45 University of Texas Southwestern Medical School, Texas, USA; 46 Emory University, Georgia, USA; 47 University of Kansas, Medical Center, Kansas, USA; 48 Mayo Clinic, Jacksonville, Florida USA; 49 Yale University School of Medicine, Connecticut, USA; 50 McGill University, Montreal-Jewish General Hospital, Quebec, Canada; 51 Sunnybrook Health Sciences, Ontario, Canada; 52 U.B.C. Clinic for AD & Related Disorders, British Columbia, Canada; 53 Cognitive Neurology-St. Joseph’s, Ontario, Canada; 54 Cleveland Clinic Lou Ruvo Center for Brain Health, Ohio, USA; 55 Premiere Research Inst (Palm Beach Neurology), Florida, USA; 56 Georgetown University Medical Center, Washington, D.C USA; 57 Stanford University, California, USA; 58 Banner Sun Health Research Institute, Arizona, USA; 59 Boston University, Massachusetts, USA; 60 Howard University, Washington, D.C USA; 61 Case Western Reserve University, Ohio, USA; 62 Neurological Care of CNY, New York, USA; 63 Parkwood Hospital, Pennsylvania, USA; 64 University of Wisconsin, Wisconsin, USA; 65 Dent Neurologic Institute, New York, USA; 66 Ohio State University, Ohio, USA; 67 Albany Medical College, New York, USA; 68 Hartford Hospital, Olin Neuropsychiatry Research Center, Connecticut, USA; 69 Dartmouth-Hitchcock Medical Center, New Hampshire, USA; 70 Wake Forest University Health Sciences, North Carolina, USA; 71 Rhode Island Hospital, Rhode Island, USA; 72 Butler Hospital, Rhode Island, USA; 73 Medical University South Carolina, Carolina, USA; 74 Nathan Kline Institute, New York, USA; 75 University of Iowa College of Medicine, Iowa, USA; 76 USF Health Byrd Alzheimer’s Institute, University of South Florida, Florida, USA

**Keywords:** Cognitive ageing, Alzheimer's disease

## Abstract

Causal Structure Discovery (CSD) is the problem of identifying causal relationships from large quantities of data through computational methods. With the limited ability of traditional association-based computational methods to discover causal relationships, CSD methodologies are gaining popularity. The goal of the study was to systematically examine whether (i) CSD methods can discover the known causal relationships from observational clinical data and (ii) to offer guidance to accurately discover known causal relationships. We used Alzheimer’s disease (AD), a complex progressive disease, as a model because the well-established evidence provides a “gold-standard” causal graph for evaluation. We evaluated two CSD methods, Fast Causal Inference (FCI) and Fast Greedy Equivalence Search (FGES) in their ability to discover this structure from data collected by the Alzheimer’s Disease Neuroimaging Initiative (ADNI). We used structural equation models (which is not designed for CSD) as control. We applied these methods under three scenarios defined by increasing amounts of background knowledge provided to the methods. The methods were evaluated by comparing the resulting causal relationships with the “gold standard” graph that was constructed from literature. Dedicated CSD methods managed to discover graphs that nearly coincided with the gold standard. For best results, CSD algorithms should be used with longitudinal data providing as much prior knowledge as possible.

## Introduction

Big data analytics, machine learning, and deep learning have garnered significant interest in the health science fields^1,2^. Due to their excellent predictive accuracy, they are increasingly employed for disease diagnosis and risk prediction^3^. However, in many biomedical applications, achieving high prediction accuracy in and by itself is not the primary goal; discovering the risk factor or mechanism that can be altered is often the primary research question.

Today’s machine learning applications are largely based on associations. Even though a risk factor may be associated with the disease, it does not necessarily mean that it can alter the disease process. In early 2018, a Phase 3 trial called “TOMMORROW” tested the effect of a diabetes drug on reducing Alzheimer’s disease (AD) dementia risk^4^. The study measured amyloid deposition, which is an early sign of Alzheimer’s disease and is also associated with diabetes. However, since diabetes is not causal to amyloidosis, the study failed in the interim analysis^5,6^. For a successful intervention, the risk factor we intervene on should have a causal (rather than merely associative) relationship with the disease outcome.

Clinical research is predominantly focused on causal relationships. Hypothesis-driven clinical research, for example, often assumes a causal structure, a set of causal relationships among biomarkers and outcomes, and researchers estimate the effect size of these relationships (e.g. causal inference). In such research, drawing a causal conclusion is valid, because prior knowledge ascertains that the relationships are indeed causal. However, when there is no knowledge of the causality, the causal structure itself needs to be discovered from data through a process known as causal structure discovery. A commonly used but incorrect practice is to assume a partial causal structure and adjust it based on output statistics of the fitted model using methods such as structural equation models (SEM).

In this work, using AD biomarkers as the predictors and cognition as the outcome, we set out to determine an optimal way to discover causal relationships. We used AD as a model for this problem because the AD biomarker cascade is well understood^7^ and the causal relationships between the primary predictors has also been well characterized such that a “gold standard” graph can be constructed. Further, the public data set of Alzheimer’s disease← neuroimaging initiative (ADNI) has extensive longitudinal data available that is conducive for the systematic comparisons planned in this manuscript. Here, we focus on comparing the results from dedicated causal discovery algorithms and a searching algorithm based on SEM, with our “gold standard” graph. We also investigated the reason behind common mistakes and explored methods to prevent them. These experiments allowed us to provide guidelines for discovering causal structure using observational data.

## Background

### Causal structure discovery algorithms

Informally, causation is defined as a relationship between two variables X and Y such that changes in X lead to changes in Y^8^. The key difference between association and causation lies in the potential of confounding. Suppose that no direct causal relationship exists between X and Y but rather a third variable Z causes both X and Y. In this case, even though X and Y are strongly associated, altering X will not lead to changes in Y. Z is called a confounder. More formally, causation is a direct effect between A and B that remains after adjusting for confounding. Confounding can be observed or unobserved (latent).

Causal structure is the set of causal relationships among a set of variables, and causal structure discovery is the problem of learning the causal structure from observational data. Dedicated causal structure discovery algorithms exist and can be separated into two subtypes, constraint-based and score-based. The constraint-based algorithms construct the causal structure based on conditional independence constraints, while the score-based algorithms generate a number of candidate causal graphs, assign a score to each, and select a final graph based on the scores. In this study, we selected one prominent algorithm from each type: Fast Causal Inference Algorithm (FCI), which is a constraint-based algorithm, and Fast Greedy Equivalence Search (FGES), which is a score-based algorithm. For brevity, we give a high-level description for FGES and FCI. For more detailed descriptions, we refer the reader to the references^9–11^. Both of the two methods can adjust for observed confounding and one of the algorithms, FCI, has some ability to discover latent confounding.

### Fast causal inference (FCI)

The central concept behind constraint-based causal discovery algorithm is the idea that different causal structures imply different independence relationships. For example, the causal relationship A→- B →C, implies that variable A is independent of C given B. On the other hand, when A →C← B, A and B are independent (unconditionally), but become dependent conditional on C. The latter structure is called the “**V**” structure (also known as collider) which has a unique independence relationship compared with other causal relationships. In fact, it is one of the “primitives” that constraint-based algorithm, like FCI, looks for.

A feature specific to FCI even among constraint-based methods is its ability to discover latent (unobserved) confounders. This is enabled by another primitive, the “**Y**” structure. Four variables define a “Y” structure when they have the following causal relationships: W1  →X ← W2 and X  →Y. Within the “Y” structure, both W1 and W2 are independent of Y conditional on X. This conditional independence helps rule out the possibility of an unmeasured confounder between X and Y. In other words, when FCI finds a “Y” structure in the graph, the causal relationship from X to Y is guaranteed to be unconfounded; otherwise, FCI assumes that possibly unobserved confounders exist^12^.

#### Fast causal inference (FCI) algorithm

FCI constructs a causal graph starting with a fully connected undirected graph, and removes edges that connect conditionally independent variables. In the second phase, it orients edges by identifying the “V” and “Y” structures, and tries to orient the remaining edges based on a set of rules which have been explained in detail elsewhere^9,13^.

#### Fast greedy equivalence search (FGES) algorithm

The Greedy Equivalence Search (GES) algorithm also has two phases. In the first phase, it starts with a graph containing no edges (corresponding to all variables being independent of each other) and greedily adds edges (dependencies) one at a time in the orientation that minimizes the Bayes Information Score^14^ (BIC), which is likelihood penalized for complexity to reduce overfitting. GES then removes edges one at a time as long as it decreases the BIC. The FGES^10^ algorithm used in this work is simply a “fast” (parallelized) version of GES^11,15^. Similarly to FCI, FGES also relies on the “V” structures to orient edges. The implied likelihood of the “V” structure is unique while the likelihoods of A →B →C, C →B →A and A ←B →C are the same. Thus, FGES will select the “V” structures when it implies a higher likelihood than other structures.

#### Structural equation modeling (SEM)

Structural Equation Modeling (SEM) is a family of statistical models, which, given the underlying causal structure, can estimate the effect size (and other statistics as well) of each relationship^16^. SEM can also suggest modifications to the given causal structure to improve model fit statistics.

While SEM was *not* designed to discover the causal structure, it is not uncommon to use SEM’s suggested modifications to “refine” the graph structure. This feature can be exploited to iteratively build a causal graph, in each iteration, adding one edge as per the suggestion by SEM. We implemented this (incorrect) searching method under two scenarios: (1) starting from the empty graph (Causal discovery); and (2) starting from a graph obtained by deleting 1 or 2 edges from the “gold standard” graph. Note that, within the scope of this paper, we use the term “SEM” to represent the algorithm that uses SEM to conduct edge searching, not to estimate the effect size.

### Key differences between the algorithms

Both SEM and FGES are stepwise algorithm which modify structure by adding or deleting edges. The biggest advantage of FGES is that it extends the search space by transforming the current structure to other “equivalent” structures. For example, given the edge A →B is in the A, B, and C graph. SEM will try adding one directed edge between C and A or C and B, yielding four possibilities. However, FGES considers more possibilities as it can also reverse the existing edge A→B to A ←B, yielding four additional possible structures.

Other than the searching strategies (Constraint versus Score based), FCI also differs from the other two algorithms in its assumption about causation: both SEM and FGES operate under the assumption of no unmeasured confounders. In other words, all the confounding variables are measured in the dataset. FCI, however, relaxes this assumption, and reports an unconfounded relationship only when it encounters a “Y” structure^17^.

**Figure 1:**
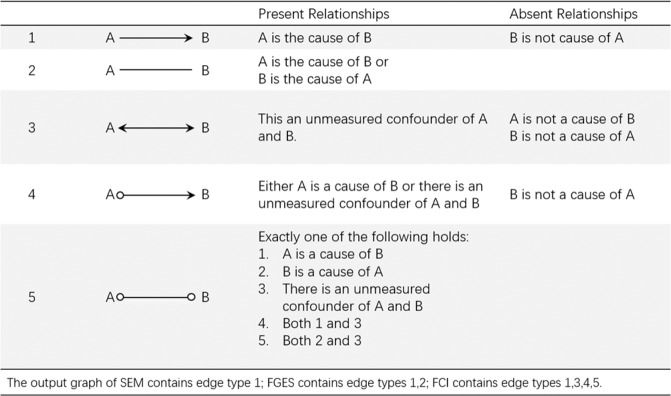
The interpretation of edges.

FCI and FGES algorithms are implemented in the Tetrad software package (Version 6.5.4). Figure 1 shows the interpretations of different edge types in the output graph. For SEM, we used the R package ‘lavaan’^18^.

## Method

### Data

Data used in the preparation of this article were obtained from the Alzheimer’s Disease Neuroimaging Initiative (ADNI) database (adni.loni.usc.edu). ADNI was launched in 2003 as a public-private partnership, led by Principal Investigator Michael W. Weiner, MD. The primary goal of ADNI has been to test whether serial magnetic resonance imaging (MRI), positron emission tomography (PET), other biological markers, and clinical and neuropsychological assessment can be combined to measure the progression of mild cognitive impairment (MCI) and early Alzheimer’s disease (AD)^19^. There are three phases of the ADNI study where the last one, ADNI3, is still ongoing. All study participants provided written informed consent, and study protocols were approved by each local site’s institutional review board. All methods were carried out in accordance with the relevant guidelines and regulations. For up-to-date information, see www.adni-info.org. IRB Review was not required since the ADNI data is de-identified and publicly available for download. We focused our study on the first two: ADNI 1 and ADNI 2/GO. The variables extracted from the data are fludeoxyglucose PET (FDG), amyloid beta (ABETA), phosphorylated tau (PTAU), apolipoprotein E (APOE) ε4 allele; demographic information: age, sex, education (EDU); and diagnosis on AD (DX). Table 1 presents summary statistics of the data set. After removing records with missing values, there are 1008 participants remaining with at least one complete record, and 266 with a regular two-year follow-up visit.

**Table 1:** Characteristics for Continuous and Categorical Variables. N = 1008.

	Label	Mean (SD)
**Demographic variables**		
AGE	AGE	74.09 (7.46)
SEX	SEX	0.55 (0.50)
Education Level	EDU	16.15 (2.71)
**Biomarkers**		
Fludeoxyglucose PET	FDG	1.22 (0.17)
Amyloid Beta	ABETA	986.29 (459.94)
Phosphorylated tau	PTAU	27.67 (14.76)
	**Label**	**subtype (%)**
**Genetics**		
APOE epsilon 4 allele	APOE4	0 (54%)/ 1 (36%)/ 2 (10%)
**Diagnosis**		
Diagnosis of Alzheimer’s Dementia	DX	CN (31%)/ MCI (46%)/ AD (23%)

### The “Gold standard” graph

**Figure 2:**
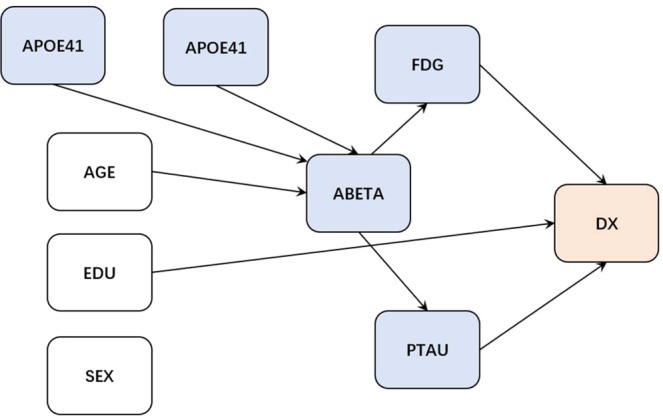
The “gold standard” graph.

The AD biomarker cascade has been evaluated widely. The deposition of ABETA in the brain is an early event in the disease process and is captured through the decrease in CSF ABETA. The only consistently shown risk factors for ABETA are age and the number of APOE4 alleles^6,20,21^. ABETA causes downstream neurofibrillary tangle formation and subsequently neurodegeneration, both of which are captured by metabolic dysfunction via FDG-PET^22^ and PTAU increase measured on CSF^23^. The two markers FDG-PET and CSF PTAU are the strongest predictors of cognitive dysfunction or diagnosis^24,25^ (in comparison to ABETA). Education, a surrogate of cognitive resilience, influences an individual’s cognitive status^26^. All of these are well established relationships in the literature. There are weaker causal associations such as sex influencing some of these associations which we did not regard to evaluate the algorithms because the impact of these associations is much smaller in comparison to the main effects considered in the “gold standard” graph. The relationships described above are shown in Fig. 2.

### Background knowledge and cross-sectional vs longitudinal data

To constrain the relationships that the algorithms can discover, background knowledge can be provided in the form of must-have or must-not-have (prohibited) edges. In this paper, we defined three degrees of background knowledge as: (Level 1) *No knowledge*: the discovered structure purely reflects the data; no edges are prohibited. (Level 2) *Trivial background knowledge*: (a) edges among demographic variables are prohibited (although association between them can remain) (b) edges from biomarkers or diagnosis to demographic variables are prohibited. (Level 3) *Longitudinal*: in addition to the edges prohibited in Level 2, edges pointing from a later time point to an earlier time point are also prohibited.

### Study design

#### Causal discovery study

We extracted two data sets from ANDI for this part of the study: one was a single cross-sectional, and data was collected at the baseline visit made by each participant. The second one is longitudinal, where we included data from two cross-sections: the baseline visit, and the visit made at the 24 months. Records with missing data were removed from further study.

To generate robust results, both the cross-sectional and the longitudinal data were bootstrapped 100 times at the participant’s level. Then, the three algorithms, SEM, FCI and FGES were tested on all bootstrap samples for evaluation, incorporating the three different degrees of knowledges that were described in the previous section.

#### SEM-recovery study

Since most researchers would start with a hypothesized graph and only use SEM to add edges, we also tested SEM under this assumed use case: we initialized (hypothesized) graphs by deleting each single edge and each pair of edges from the “gold standard” graph, and then tested whether SEM can recover the deleted edges after no more than five iterations of edge adding. We chose five in this study because more than five times of edge adding will result in a graph with low recall.

### Evaluation metrics

To assess the performance of methods, we defined following evaluation metrics. An edge is correct, if and only if the same edge exists in the “gold standard” graph and the orientation of the edge coincides with the orientation in the “gold standard” graph; an edge is semi-correct, if and only if the same edge exists in the “gold standard” graph and its orientation does not contradict with the true orientation of the edge in the “gold standard” graph; And finally, an edge is incorrect if the edge does not exist in the “gold standard” graph or if it exists but its orientation is the opposite of the true orientation.

We will present the following metrics:

1. Number of correct, semi-correct, incorrect edges
2. Precision: the proportion of correct or semi-correct edges over all edges reported by the algorithm
3. Recall: the proportion of edges in the “gold standard” graph that are correctly or semi-correctly reported
4. Occurrence rate: the percentage of the adjacency shows in the result of the 100 bootstrap runs

## Results

### Causal discovery study

The discovered causal structures generated by SEM, FCI, and FGES algorithms across three degrees of prior “knowledge” are shown in this section. The behind-the-scenes mechanism of typical mistakes will be examined further in the discussion section.

#### Experiment 1: Without background knowledge

**Figure 3:**
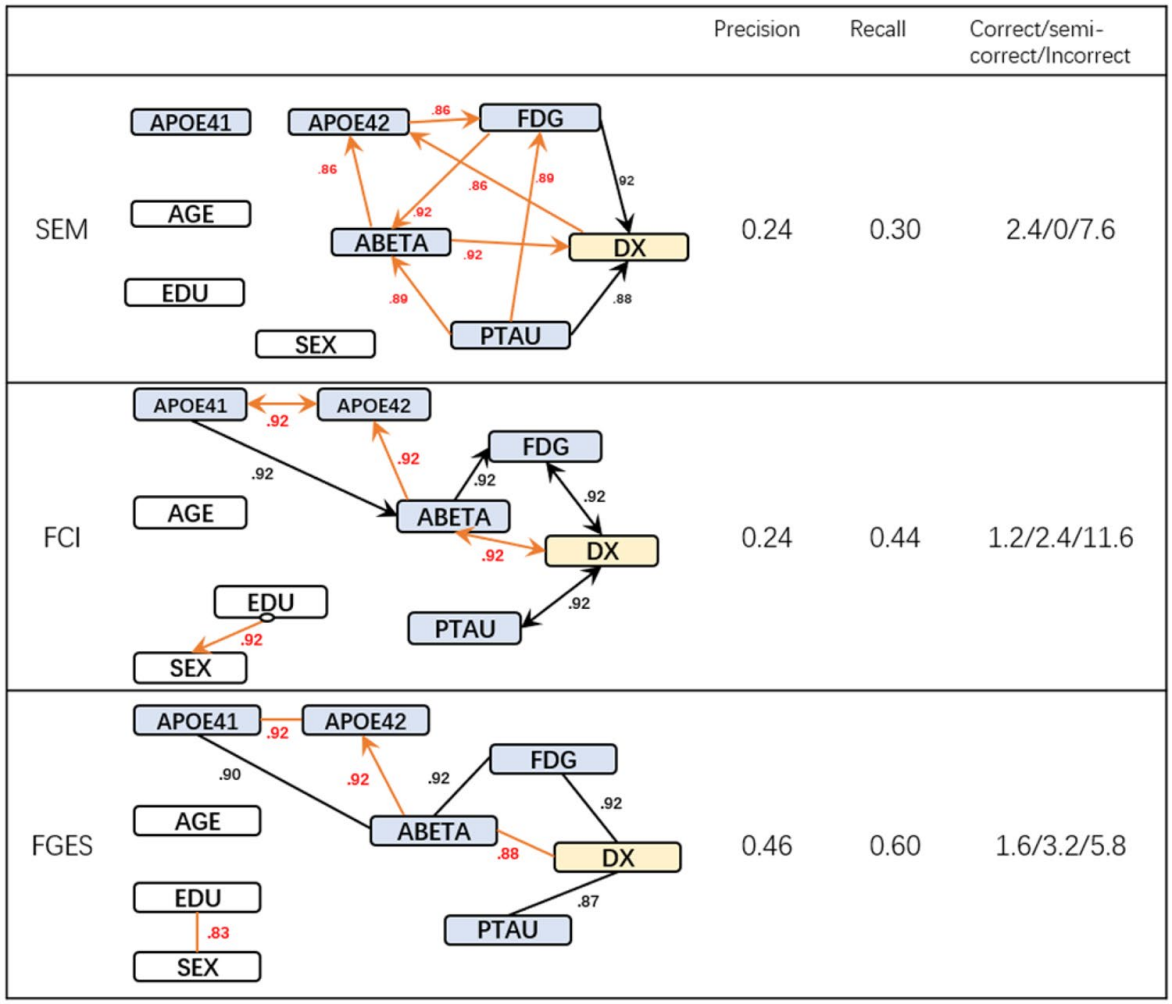
Discovered causal structure without background knowledge & their Statistics.

In Fig. 3, we present the edges with at least 80% occurrence rate in the 100 bootstraps samples (The number located near each edge). The edges that were not in the gold standard graph are colored in red. The numbers on the right of each graph are the precision, recall, and the number of correct, semi-correct, and incorrect edges averaged over the 100 bootstrap samples. To ease direct comparison, the variables are laid out almost identically: the same variable occupies the same relative location in all three graphs.

SEM was only able to retrieve two correct edges from the “gold standard” graph (with average precisions 0.24 and recall 0.30), while FCI and FGES found 4 out of 8 edges correctly or semi-correctly (precision 0.24 and 0.44, recall 0.46 and 0.6 correspondingly). Both FCI and FGES successfully recovered the causal relationship between genetic variable APOE41 with ABETA, ABETA with FDG, and FDG, PTAU with DX. However, the algorithms failed to determine the directionalities of some of the relationships. We also observed that all three algorithms reported edges from biomarkers to demographic variables which are certainly errors (e.g. ABETA causes APOE42 in FCI’s graph). It is important to note that some well-established relationships such as age and amyloid as well as education and diagnosis were not discovered in any of the graphs which did not have background knowledge.

#### Experiment 2: Addition of trivial background knowledge

**Figure 4:**
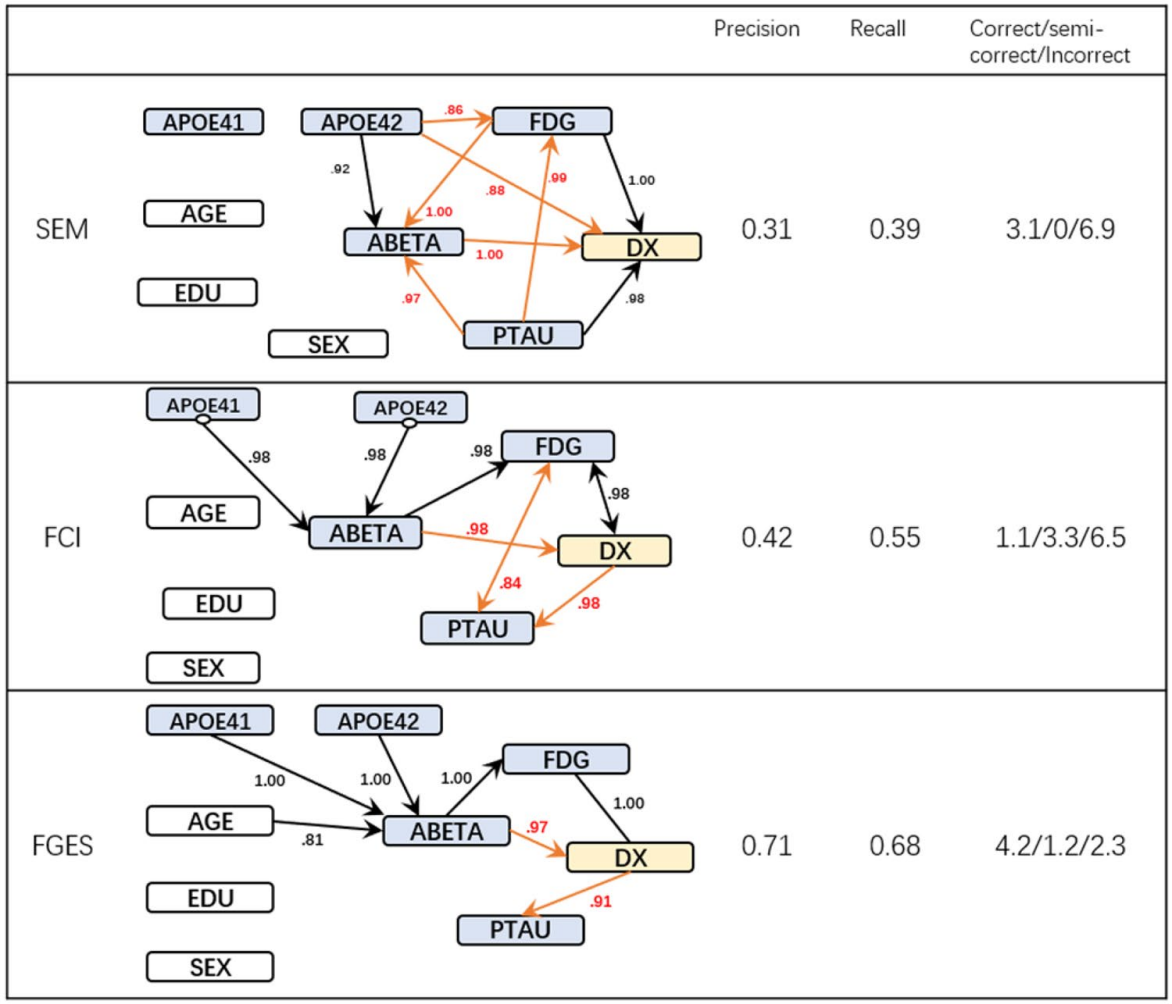
Discovered causal structure with background knowledge & their Statistics.

Figure 4 presents the causal structures discovered by the three algorithms incorporating trivial background knowledge: demographic variables cannot be caused by other demographic variables nor by biomarkers (e.g. participant’s age is not affected by education or ABETA level). The structure of Fig. 4 is analogous to Fig. 3.

While all the methods made several mistakes, there were significant improvements when trivial background information was added. Some of the incorrect causations found by SEM are actually indirect causal relationships in the “gold standard”. For example, the effect from APOE42 to DX is an indirect effect that flow through ABETA in the ‘gold standard’ graph. All three algorithms discovered the edge ABETA→DX. Though it is not a direct casual effect in our “gold standard” graph, ABETA is a common cause of FDG reduction as well as PTAU increase and both FDG and PTAU lead to DX. Therefore, the effect of ABETA and DX could be anticipated. Both FCI and FGES reported a falsely-directed edge between PTAU and DX. We will see this error is corrected by using longitudinal data. The FCI algorithm also reported unmeasured confounders between PTAU and DX with FDG, which are interesting hypotheses that need further studies. Among the three algorithms, SEM achieved the lowest performance (Precision 0.31, recall 0.39) while FCI and FGES achieved higher and substantial higher performance (FCI: 0.42 precision and 0.55 recall; FGES 0.71 precision and 0.68 recall).

#### Experiment 3: Addition of longitudinal data and trivial background knowledge

**Figure 5:**
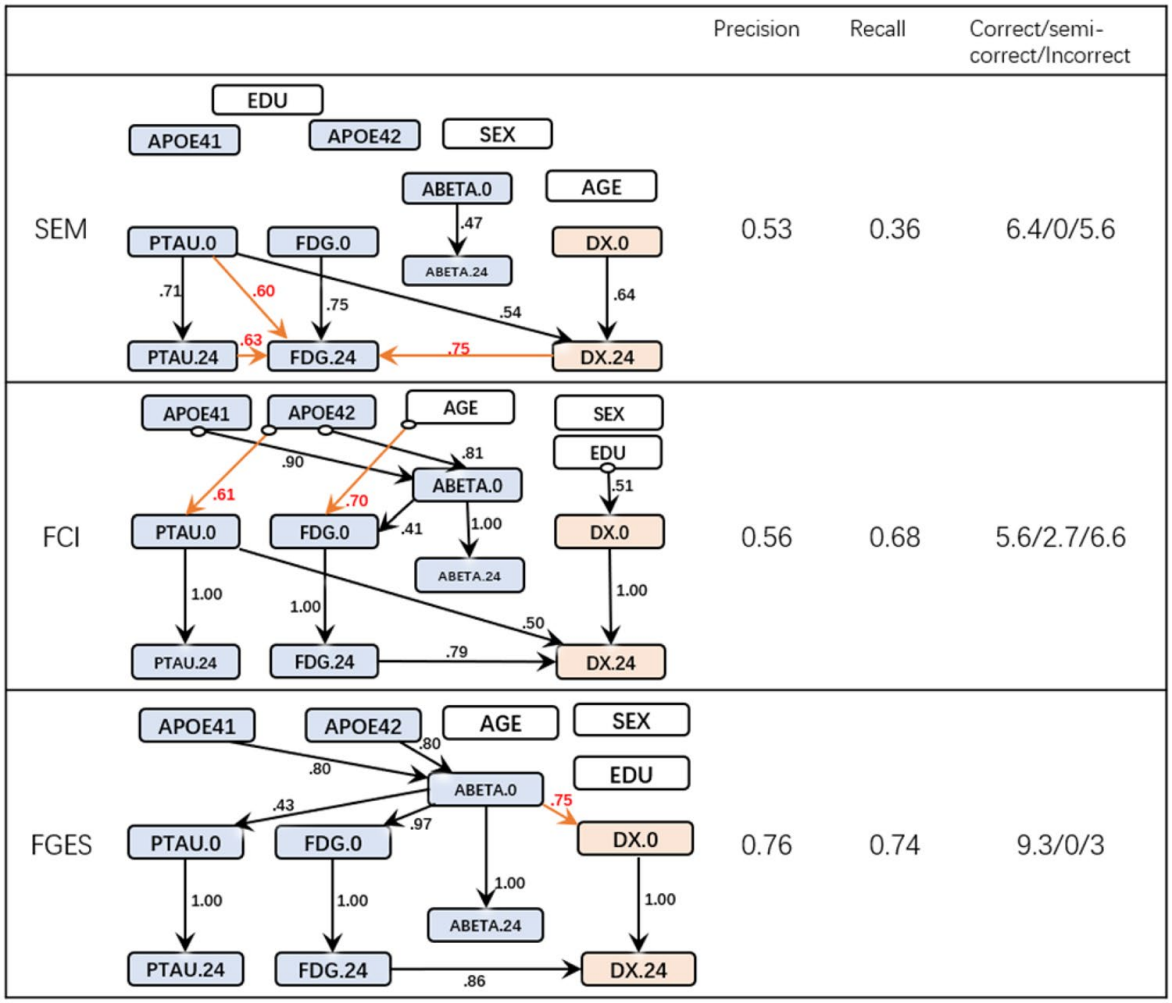
Discovered causal structure with longitudinal data & their Statistics.

Figure 5 presents the most frequent edges discovered by the three algorithms and their performance metrics. The layout of the graphs is different from the previous Figures because they are built on the longitudinal data set. All nodes associated with biomarkers or diagnosis hence appear twice: once with their baseline value (denoted by ‘0.0’ suffix) and once at 24 months (denoted by the ‘0.24’ suffix). Most of the statistics further improved relative to previous results and FGES recovered a graph with only one incorrect edge.

The performance of the SEM algorithm trailed behind the other two dedicated causal discovery algorithms. In some of the bootstrap runs, SEM missed the direct effects between the longitudinal measurements of the same biomarker (e.g. the estimated probability of SEM discovered the edge ABETA.0 →ABETA.24 is 0.47 where FCI and FGES always include this edge).

The FCI algorithm further identified that PTAU at initial visit has an effect on the diagnosis (AD) at 24 months. In other words, PTAU may have a lagged effect on AD diagnosis which is a highly plausible hypothesis. We also observed that AGE and EDUCATION lead to different FDG and diagnosis at the first visit, but not directly to the assessment at 24 months (after adjusting the assessment at baseline visit).

The FGES algorithm incorporated the longitudinal data and successfully discovered the FDG to DX edge. It also removed the incorrect edges from DX to PTAU. Furthermore, with longitudinal data, the previously undirected edges identified by FGES got directed without compromising the overall precision and recall.

### SEM-recovery study

Table 2 shows the statistics when we tested SEM’s ability to recover deleted edges from the “gold standard” graph. In each run, we deleted a single edge or a pair of edges. The “fully recovery rate” represents the percentage of runs in which SEM managed to fully recover the deleted edge(s). The “precision” and “recall” columns are defined the same way as in the previous experiments. As we can see from Table 2, when we removed only a single edge, the recovery rate is very low (12.5%). When we removed two edges from the “gold standard” graph, SEM was unable to recover the true graph.

**Table 2:** Recovery rate of edges.

Number of edges removed	Fully recover rate	Precision. Mean	Recall. Mean
1	0.125	0.67	0.89
2	0	0.70	0.76

## Discussion

In this study, we compared three different methodologies to recreate the known ground-truth causal structure based on an observational dataset using three different degrees of “knowledge”. We used Alzheimer’s disease data from ADNI which is a well characterized openly accessible data set. Since the relationships among biomarkers and diagnosis in Alzheimer’s disease are well understood, we began with a “gold-standard” causal structure based on the existing literature. Then, we applied three algorithms to discover this causal structure from data. This work highlights the common errors made by the different algorithms and offers us with ideas and suggestions to avoid these errors. In the end, a detailed guideline on how causal discovery algorithm can be applied to discover high-quality causal relationships was provided.

Each of the three algorithms used in this work represents a class of algorithms with its specific characteristics. Two of the algorithms, FCI and FGES, are dedicated causal discovery algorithms, while the third one, SEM, is primarily designed as a confirmatory tool. The dedicated causal discovery algorithms outperformed SEM across all three degrees of background knowledge. This is not surprising, because SEM is not specifically designed to discover causal structure; statistics reported by SEM only indicate possible adjustments to the a priori user-defined causal structure. What is surprising is the extent to which FGES outperformed SEM, since both FGES and SEM optimize the same criterion, which is BIC. The key difference between FGES and SEM is the scale of the underlying search space: FGES considers a broader array of graphs, all graphs that have the same dependence structure (same set of conditional independence relationships among the variables). From the SEM-recovery experiment, we also observed that the SEM’s suggestions for adding edges are generally not reliable. These edges may maximize BIC in SEM’s limited search space, but these are not the overall optimal edges: FGES, with its larger search space, managed to (almost perfectly) recover the “gold standard” graph.

With FCI and FGES having similar search spaces, the main differences between them lies in their search algorithm. The performance of the score-based algorithm FGES was higher and was more stable than the constraint-based algorithm FCI in our study. The decision making of FCI was affected by the incorrect independence tests introduced by selection bias or data artifacts. These mistakes propagated to other parts of the graph through generating incorrect “V” or “Y” structures and eventually caused damage to large portions of the graph. In contrast, score-based algorithms consider the likelihood of the global structure while making local decisions; so, these errors remain localized. This explains why the discovered structure of FGES before or after adding trivial knowledge are more consistent. FCI has the advantage of being able to relax the typical assumption of no unmeasured confounders. This relaxation can be useful when either identifying unmeasured confounders or finding unconfounded causal relationships is important. In our study, FCI found that the relationship from ABETA to FDG is un-confounded and that unmeasured confounders may exist between FDG, DX (Fig. 4-FCI)

We further investigated the mistakes that FCI and FGES made. We grouped these mistakes into three categories and described their causes and work-arounds in Table 3.

1. The first kind of error happens when artifacts in the data induce incorrect edges. For example, FCI reported an edge between EDU and SEX (Fig. 3-FCI), because in our sample, the average education level of male participants is higher. Avoiding such incorrect edges is important because they can potentially create incorrect “V” or “Y” structures that jeopardizes the remaining causal discovery steps. Adding trivial background knowledge can resolve this problem by preventing the algorithms from treating association as causation.
2. When universally accepted background knowledge is not available, compensating for data artifacts is more difficult and can have distant downstream effects. For example, the APOE42-PTAU-FDG structure was inferred as a “V” structure (APOE42 →PTAU ←FDG) in some of the bootstrap runs. This error was a result of a single incorrectly inferred independence between APOE42 and FDG from the sample data. This error propagated through three associations between (1) APOE42 and PTAU, (2) PTAU and FDG, and (3) APOE42 and FDG conditional on PTAU, which led to a “V” structure among the three. We cannot prevent this edge using background knowledge unless we know the conditional independence relations between APOE42 and FDG beforehand. The use of longitudinal data helped correct these mistakes; as we can see in Fig. 5-FCI, the substructure was corrected when longitudinal data was used.
3. While longitudinal data provides a solution to a number of the problems, the requirement of repeated observations can constrain the sample size and introduce some errors of its own. For example, FCI discovered a possible lagged relationship between PTAU at time 0 and DX at month 24 (Fig. 5-FCI), however, the same relationship is not observed in the results from FGES (Fig. 5-FGES)–possibly due to the small sample size.

**Table 3:** Typical problems and solutions.

	Error	Location	Reason for error	Solution
1	EDU and SEX	Fig. 3-FCI	Selection bias	Add trivial knowledge
2	APOE4, PTAU and FDG	Fig. 4-FCI	Selection bias or Artifacts and No background knowledge	Longitudinal data
3	PTAU → DX	Fig. 5-FGES	Small sample size	Increase sample

In the longitudinal study, local structure across time points are not guaranteed to be the same. For example, in the graph discovered by FGES, ABETA.0 causes PTAU.0 but ABETA.24 does not cause PTAU.24. The reason is that ABETA.0 is a common parent of ABETA.24 and PTAU.0. PTAU.24 is conditionally independent of ABETA.24 given either ABETA.0 or PTAU.0. This conditional independence relationship implies that in the presence of ABETA.0 or PTAU.0, ABETA.24 is not needed to explain the variation in PTAU.24.

Even though the final graph learnt from observational data matched the “gold standard” graph closely, our conclusions still depend on the correctness of the “gold standard”. In general, we have high confidence in the “gold standard” graph as the biological mechanism behind the AD biomarker cascade is well understood and FGES managed to discover the “gold standard” almost perfectly. However, it is highly possible that FDG and PTAU only explain part of the effect from ABETA on diagnosis of AD; a direct causation from ABETA on DX could exist. Although we recommend longitudinal data, collecting data longitudinally is often costly which typically results in a smaller sample size. Small sample size lowers the statistical power in causal discovery algorithm which is a trade-off. We tried reducing the sample size by 50% and 75% and conducted the same analysis. When we reduced the sample size by 50%, the total numbers of discovered edges across 100 bootstrap iterations reduced. However, edges that were consistently discovered on the full sample were consistently discovered on the reduced sample as well. We achieved similar precision and recall. When we further reduced the sample size (by a total of 75%), the total number of edges further reduced. While the most frequently discovered edges continued to get discovered, the number of “noise edges”, edges that were discovered only in a few bootstrap iterations, increased.

In conclusion, dedicated causal discovery algorithms outperformed SEM in discovering the causal structure. In real-world data analysis, data quality impacted the correctness of the discovered structure. Incorporating prior knowledge and using longitudinal data can improve the discovered result by preventing algorithms from make some potential mistakes.
